# Functional Characterization of the FNT Family Nitrite Transporter of Marine Picocyanobacteria

**DOI:** 10.3390/life5010432

**Published:** 2015-02-09

**Authors:** Shin-ichi Maeda, Akio Murakami, Hisashi Ito, Ayumi Tanaka, Tatsuo Omata

**Affiliations:** 1 Graduate School of Bioagricultural Sciences, Nagoya University, Nagoya 464-8601, Japan; E-Mail: omata@agr.nagoya-u.ac.jp; 2 CREST, Japan Science and Technology Agency, Chiyoda-ku, Tokyo 102-0076 Japan; E-Mails: akiomura@kobe-u.ac.jp (A.M.); ito98@lowtem.hokudai.ac.jp (H.I.); ayumi@pop.lowtem.hokudai.ac.jp (A.T.); 3 Research Center for Inland Seas, Kobe University, Awaji, Hyogo 656-2401, Japan; 4 Institute of Low Temperature Science, Hokkaido University, Kita-ku, Sapporo 060-0819, Japan

**Keywords:** marine cyanobacteria, nitrite, transporter

## Abstract

Many of the cyanobacterial species found in marine and saline environments have a gene encoding a putative nitrite transporter of the formate/nitrite transporter (FNT) family. The presumed function of the gene (designated *nitM*) was confirmed by functional expression of the gene from the coastal marine species *Synechococcus* sp. strain PCC7002 in the nitrite-transport-less mutant (NA4) of the freshwater cyanobacterium *Synechococcus elongatus* strain PCC7942. The NitM-mediated nitrite uptake showed an apparent *K*_m_ (NO_2_^−^) of about 8 μM and was not inhibited by nitrate, cyanate or formate. Of the *nitM* orthologs from the three oceanic cyanobacterial species, which are classified as α-cyanobacteria on the basis of the occurrence of Type 1a RuBisCO, the one from *Synechococcus* sp. strain CC9605 conferred nitrite uptake activity on NA4, but those from *Synechococcus* sp. strain CC9311 and *Prochlorococcus* *marinus* strain MIT9313 did not. A strongly conserved hydrophilic amino acid sequence was found at the C-termini of the deduced NitM sequences from α-cyanobacteria, with a notable exception of the *Synechococcus* sp. strain CC9605 NitM protein, which entirely lacked the C-terminal amino acids. The C-terminal sequence was not conserved in the NitM proteins from β-cyanobacteria carrying the Type 1b RuBisCO, including the one from *Synechococcus* sp. strain PCC7002. Expression of the truncated *nitM* genes from *Synechococcus* sp. strain CC9311 and *Prochlorococcus* *marinus* strain MIT9313, encoding the proteins lacking the conserved C-terminal region, conferred nitrite uptake activity on the NA4 mutant, indicating that the C-terminal region of α-cyanobacterial NitM proteins inhibits the activity of the transporter.

## 1. Introduction

Contribution of marine primary producers to global net primary productivity is comparable to that of terrestrial primary producers [1]. Although the larger part of net primary productivity of the sea is contributed by eukaryotic algae living in nutrient-rich regions, 22% of the net primary production is carried out in the oligotrophic environments of the tropical and subtropical oceans [1]. The supply of nitrogen to marine phytoplankton in nutrient-rich region is largely through the uptake of nitrate and ammonium, although dissolved organic nitrogen and dissolved dinitrogen gas significantly contribute to nutrition of marine plankton [2,3,4,5]. However, concentrations of nitrate and ammonium are limited in most regions of the tropical and subtropical oceans and nitrogen availability has an important role in net primary productivity in these regions [6,7]. Picoplanktonic cyanobacteria of *Synechococcus* and *Prochlorococcus* strains are often the numerically dominant phytoplankton group and an important contributor to the net primary production in the tropical and subtropical oceans [8].

In the tropical Atlantic Ocean, ammonium concentration is limited (~0.05 µM). Nitrate concentration is dependent on the depth, being low (<0.01 µM) in the upper layer and high (>10 µM) in the deeper layer (>100 m). Nitrite (~0.6 µM) is present only in the middle layer (~60 m) [5]. In the tropical Pacific Ocean, similar ion distribution was observed [9] and the distribution of the chlorophyll (Chl) density was similar to that of nitrite concentration (Website of Japan Meteorological Agency [10,11]), suggesting that there are the ocean areas where nitrite is the important nitrogen source for the phytoplankton.

Nitrate and nitrite are transported into cyanobacterial cells by active transport system(s). Nitrate is reduced to nitrite by nitrate reductase and nitrite is further reduced to ammonium by nitrite reductase. The resulting ammonium is assimilated as the amide group of Gln by glutamine synthetase. All cyanobacteria have the *glnA* gene encoding glutamine synthetase and can assimilate ammonium. While all the marine *Synechococcus* strains have the *narB* gene encoding nitrate reductase as well as the *nirA* gene encoding nitrite reductase and have the capacity to assimilate both nitrate and nitrite, most *Prochlorococcus* strains lack the *narB* gene. Many of these *Prochlorococcus* strains lack *nirA* as well, but there are a number of strains retaining *nirA* [12,13,14]. The occurrence of the *nirA* carrying *Prochlorococcus* strains support the notion that nitrite could be an important nitrogen source in the tropical ocean.

Badger *et al.* advocated that two primary groups of cyanobacteria could be distinguished based on their RuBisCO phylogeny [15]. These two groups also have distinct sets of proteins constituting their carboxysomes. Thus the first of the two groups, having the type 1A RuBisCO and α*-*carboxysomes, is referred to as α*-*cyanobacteria and the other, having the type 1B RuBisCO and β-carboxysomes, is referred to as β-cyanobacteria. The group of α*-*cyanobacteria consists of the marine picoplanktonic *Synechococcus* and *Prochlorococcus* strains and the species of the *Cyanobium* genus. Although *Cyanobium* seems to include freshwater strains, all the other α*-*cyanobacteria characterized to date are marine strains. Including filamentous and diazotrophic strains, β-cyanobacteria are more diverse than *α-*cyanobacteria and a large variety of β-cyanobacteria comprise the group of freshwater cyanobacteria. It should be noted, however, that β-cyanobacteria also include marine strains, e.g., *Synechococcus* sp. strain PCC 7002 and *Trichodesmium erythraeum* IMS101, and halotolerant strains, e.g., *Dactylococcopsis salina* strain PCC 8305 and *Halothece* sp. strain PCC 7418.

In addition to the nitrate and nitrite reductases, an active transport system for nitrate is required for assimilation of nitrate. There are two types of high-affinity nitrate transporters in cyanobacteria: the ABC transporter encoded by the *nrtABCD* genes [16,17,18] and the MFS family transporter encoded by the *nrtP* gene [19]. While the freshwater cyanobacteria have either or both of the two transporters, the strains from marine and saline environments, including both the α- and β-cyanobacteria, generally have NrtP as the sole species of nitrate transporter, suggesting that the pattern of distribution of the nitrate transporters has been determined by environmental conditions rather than by taxonomic positions. Another nitrogen related feature of marine and halotolerant strains is the occurrence of a gene coding for a putative nitrite transporter of the formate/nitrite transporter (FNT) family. This gene is found in most of the marine α-cyanobacterial strains capable of nitrite assimilation and a few of the β-cyanobacteria from marine and saline environments, but is rarely found in freshwater cyanobacteria. The gene is presumed to encode a nitrite transporter because the gene is tightly linked with the nitrite reductase structural gene *nirA* in *α*-cyanobacteria and that the encoded protein is similar to the FNT family nitrite transporters from various organisms, including the NAR1 protein of the green alga *Chlamydomonas reinhardtii* [20], the NitA protein of the fungus *Aspergillus nidulans* [21], and the NirC protein of *Escherichia coli* [22], although a functional characterization of the gene is yet to be performed.

In this study, we verified the nitrite transport activity of the FNT family proteins from four strains of marine cyanobacteria through functional expression of the protein in the nitrite-transport-less mutant of the freshwater cyanobacterium *Synechococcus elongatus* strain PCC 7942. It is shown that the transporters from *α*-cyanobacteria have a regulatory domain that inhibits the transport activity. 

## 2. Results

### 2.1. Distribution of the Gene Encoding an FNT Family Protein in Cyanobacteria

**Figure 1:**
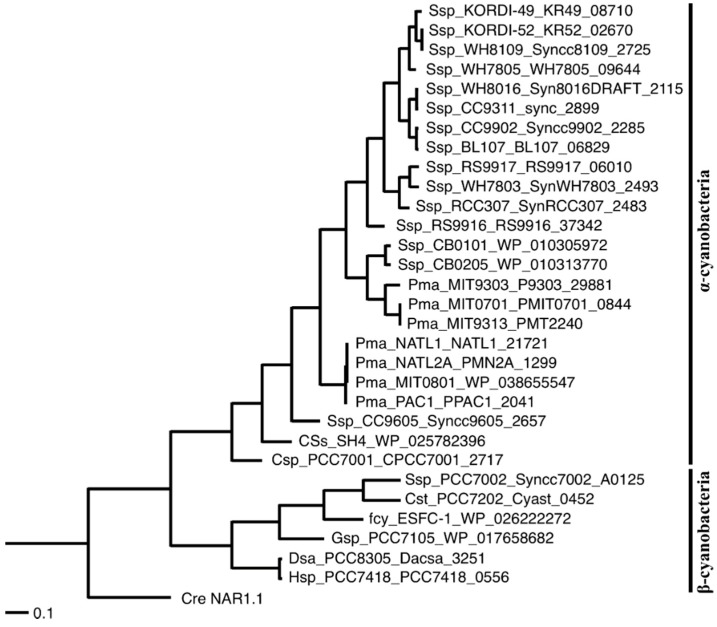
Phylogenetic tree of the NitM proteins of cyanobacteria. The NAR1.1 protein of the green algae *Chlamydomonas reinhardtii* was added as outgroup. Phylogenies are based on protein sequences that were aligned using the program ClustalX. A phylogenetic tree was created using the UPGMA (Unweighted Pair Group Method with Arithmetic mean) clustering method of ClustalX. The abbreviations for the species names are as follows. Cre, *Chlamydomonas reinhardtii*; Csp, *Cyanobium* sp.; CSs, *Candidatus* Synechococcus spongiarum; Cst, *Cyanobacterium stanieri*; Dsa, *Dactylococcopsis salina*; fcy, filamentous cyanobacterium; Gsp, *Geitlerinema* sp.; Hsp, *Halothece* sp.; Pma, *Prochlorococcus marinus*; Ssp, *Synechococcus* sp.

As of September 2014, there are 72 completely sequenced cyanobacterial genomes available in GenBank, 21 of which are from α-cyanobacteria and the other 51 genomes are from β-cyanobacteria (Supplementary Table S1). Among the α-cyanobacterial strains, seven strains have the capacity of nitrate assimilation and eleven have the capacity of nitrite assimilation, with nine of the eleven strains carrying the gene for the putative nitrite transporter of the FNT family. The gene is closely linked to the nitrite reductase gene *nirA* in α-cyanobacteria, suggesting that the gene is associated with the capacity for nitrite assimilation. Unlike α-cyanobacteria, β-cyanobacteria are generally capable of assimilation of nitrate and nitrite, but only four out of the 51 β-cyanobacteria were found to have the gene encoding the FNT family proteins. These include the coastal marine strain *Synechococcus* sp. strain PCC 7002 and the two halotolerant strains *Dactylococcopsis salina* strain PCC 8305 and *Halothece* sp. strain PCC 7418. It thus appeared that the FNT family protein is related to nitrite utilization in marine and saline environments. Phylogenetic analysis of all of the FNT family proteins available in GenBank show that the proteins from α-cyanobacteria and β-cyanobacteria form distinct groups (Figure 1).

### 2.2. Characterization of the FNT Family Transporter from Synechococcus sp. Strain PCC 7002

**Figure 2:**
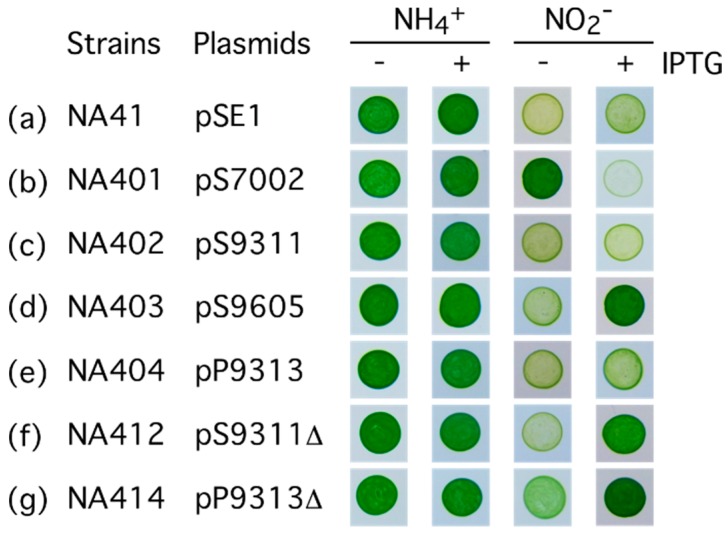
Growth test on nitrite-containing media showing the effects of expression of the genes encoding the FNT family proteins of marine cyanobacteria in the NA4 mutant lacking the ABC-transporters capable of nitrite transport. *Synechococcus elongatus* strain PCC 7942 cells (*n* = 10^6^) were spotted onto solid medium containing 7.5 mM ammonium or 0.5 mM nitrite and incubated under illumination for four days. The medium containing ammonium was buffered at pH 8.2 and the medium containing nitrite was buffered at pH 9.6. Where indicated, isopropyl-β-d-thiogalactopyranoside (IPTG; 1 mM) was added to induce the expression of the plasmid-borne genes.

**Figure 3:**
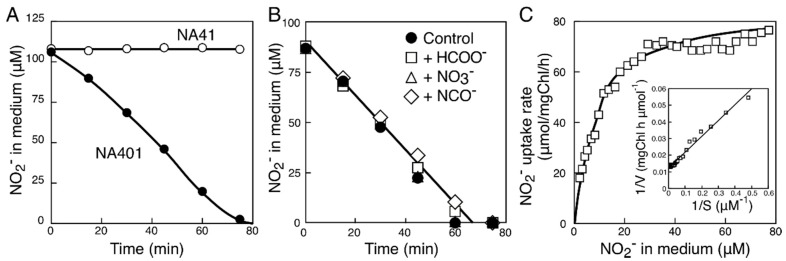
Uptake of nitrite from medium by cells of NA401 carrying pS7002. (**A**) Changes in the nitrite concentration in the medium after the addition of nitrite to cell suspensions of NA41 carrying pSE1 (control, open circles) and NA401 (closed circles) containing 1 μg of Chl/mL; (**B**) Effects of oxoanions on the uptake of nitrite by cell suspensions of NA401 containing 1 μg of Chl/mL. Nitrite was added at time zero to the cell suspensions, with simultaneous addition of 1 mM formate (open squares), 1 mM nitrate (open triangles), or 1 mM cyanate (open diamonds); (**C**) Effect of nitrite concentration on the rate of nitrite uptake. The rate of nitrite uptake was obtained by calculating the difference of the nitrite concentration in the medium measured at 5-min intervals using cell suspensions of NA401 containing 0.5 μg of Chl/mL. *Inset*, Double-reciprocal plot of the data.

In our study on the putative nitrite transporter genes of marine cyanobacteria, we first examined the function of the gene from the β-cyanobacterium *Synechococcus* sp. strain PCC 7002 (*synPCC7002_A0125*). A transcriptional fusion of the *trc* promoter and *synPCC7002_A0125* was introduced into the NA4 mutant to construct the NA401 strain by using the pSE1 shuttle expression vector [23]. The pSE1 vector was also introduced into NA4 to construct the reference strain NA41. Both strains grew well on the medium containing ammonium (Figure 2a,b). Whereas NA41 failed to grow on the nitrite (0.5 mM)-containing medium of pH 9.6 irrespective of the presence of IPTG (Figure 2a), NA401 grew well on the medium in the absence of IPTG (Figure 2b). In the presence of IPTG, NA401 died on the nitrite-containing medium but grew well on the ammonium-containing medium (Figure 2b). These results suggested that the basal-level expression of *synPCC7002_A0125* from the *trc* promoter was sufficient to support nitrite uptake from the medium containing low concentrations of nitrite, but overexpression of the gene resulted in uptake of excessive nitrite from the medium to kill the cells. To further analyze the role of the SynPCC7002_A0125 in nitrite transport, nitrite uptake activity of these mutants was determined by measuring consumption of nitrite in liquid medium (Figure 3). Whereas NA41 failed to utilize low concentrations of nitrite, the non-induced cells of NA401 without IPTG treatment took up low concentrations of nitrite (Figure 3A). Nitrite uptake rate of the cells of NA401 was calculated to be 80 µmol (mg·Chl)^−1^·h^−1^ in the extracellular nitrite concentration range of 20–100 µM. These results confirmed that the FNT family protein of *Synechococcus* sp. strain PCC 7002 has nitrite uptake activity. We therefore named the gene as *nitM* for nitrite transporter of marine cyanobacteria. When grown under the same conditions, the wild-type cells of *S. elongatus* strain PCC 7942, expressing the ABC-type bispecific nitrate/nitrite transporter, showed a rate of about 40 µmol (mg Chl)^−1^·h^−1^ for the uptake of nitrate or nitrite [23]. Thus, the nitrite uptake rate of the non-induced NA401 cells was two fold of that of the wild-type *S. elongatus* strain PCC 7942 cells.

In *S. elongatus* strain PCC 7942, nitrite uptake by the two ABC transporters capable of nitrite transport is inhibited by their alternative substrates, *i.e.*, nitrate and cyanate, respectively [23,24]. Nitrite uptake by NA401, by contrast, was not affected by nitrate or cyanate added at a concentration 10-fold higher than that of nitrite (Figure 3B). Nitrite uptake by NA401 was not affected by formate, either (Figure 3B). These results suggested that the NitM protein of *Synechococcus* sp. strain PCC 7002 specifically transports nitrite. The rate of nitrite uptake by NA401 followed a saturation-type kinetics with respect to the nitrite concentration in medium with a *K*_m_ (NO_2_^−^) of 8 µM and a *V*_max_ of 83 µmol (mg Chl)^−1^·h^−1^ (Figure 3C). The affinity for nitrite of the transporter was slightly lower than those of the NAR1 protein of *Chlamydomonas reinhardtii* (*K*_m_ = 5 µM) [20] and the NitA protein of *Aspergillus nidulans* (*K*_m_ = 4 µM) [21].

### 2.3. Characterization of the NitM Proteins from α-Cyanobacteria

**Figure 4:**
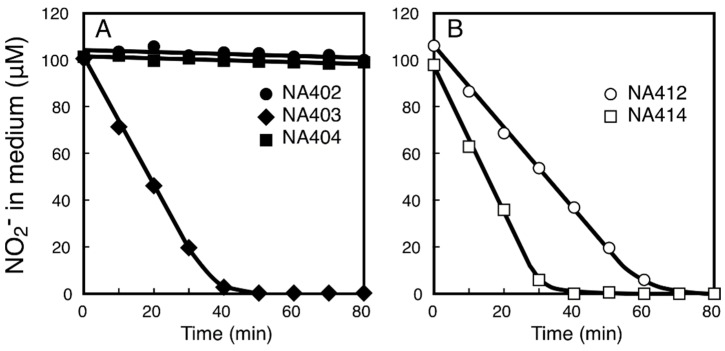
Uptake of nitrite from medium by cells of the NA4 derivatives expressing the NitM proteins and their derivatives from α-cyanobacteria. Changes in the concentration of nitrite in the medium after addition of nitrite to cell suspensions containing 5 μg of Chl/mL are shown. (**A**) NA402 (closed circles), NA403 (closed diamonds), and NA404 (closed squares) cells; (**B**) NA412 (open circles) and NA414 (open squares) cells. Cells grown in the presence of 1 mM isopropyl-β-d-thiogalactopyranoside were used for the measurements.

As *Synechococcus* sp. strain PCC 7002 belongs to β-cyanobacteria, we further characterized the *nitM* genes of the oceanic α-cyanobacterial strains. Transcriptional fusions of the *trc* promoter and the *nitM* genes of *Synechococcus* sp. strain CC9311 (sync_2899), *Synechococcus* sp. strain PCC9605 (Syncc9605_2657) and *Prochlorococcus marinus* strain MIT9313 (PMT2240) were introduced into the NA4 mutant of *S. elongatus* to construct the NA402, NA403 and NA404, respectively. On the medium containing 0.5 mM nitrite at pH 9.6, IPTG-induced cells of NA403 grew well (Figure 2d), while the NA402 and NA404 mutants failed to grow irrespective of the presence or absence of IPTG (Figure 2c,e). In accordance with the results of the growth experiment, IPTG-treated NA403 cells took up low concentrations of nitrite from the medium of pH 9.6 (Figure 4A), while the NA402 and NA404 strains failed to utilize low concentrations of nitrite. Thus the NitM protein of *Synechococcus* sp. strain PCC9605 was functional as a nitrite transporter when expressed in the *S. elongatus* NA4 mutant, but those from *Synechococcus* sp. strain CC9311 and *Prochlorococcus marinus* strain MIT9313 were not.

**Figure 5:**
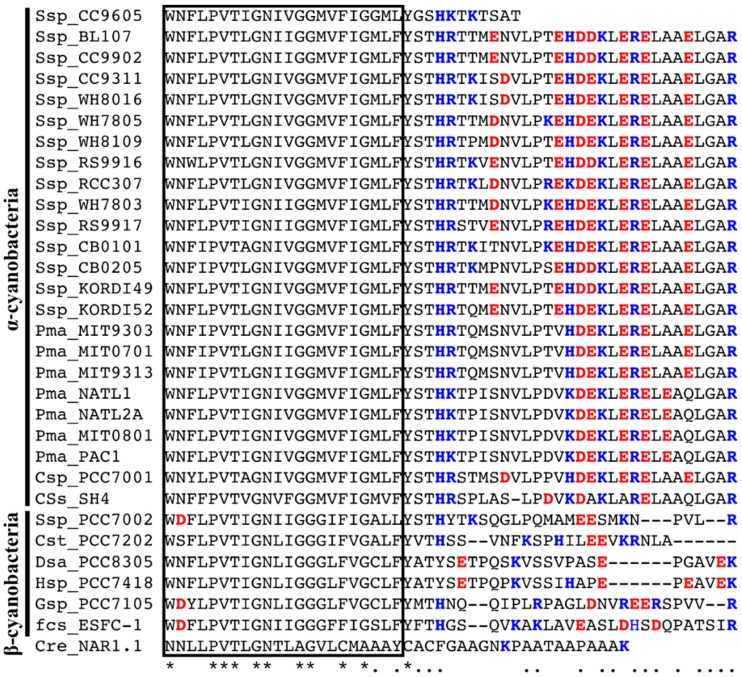
Alignment of the amino acid sequences of the C-terminal region of the cyanobacterial NitM proteins and the NAR1 protein from *Chlamydomonas reinhardtii*. Positively and negatively charged amino acid residues are indicated by blue and red characters, respectively. The Box indicates the sixth predicted transmembrane region. The source organisms are same as Figure 1. Asterisks indicate the amino acid residues common to all the NitM protein sequences. Dots indicate the amino acid residues common to all the α-cyanobacterial NitM proteins but the one from *Synechococcus* sp. strain CC9605. Dashes indicate gaps introduced to enhance the alignment.

Among the four NitM proteins functionally characterized above, those from *Synechococcus* sp. strains CC9605 and CC9311 showed the strongest similarity to each other, being 82% identical to each other. The only prominent difference between the two sequences was that NitM of strain CC9605 was shorter by 20 amino acids than that of strain CC9311. Comparison of all the NitM sequences available in GenBank showed that all the NitM proteins of α-cyanobacteria, excluding the one from *Synechococcus* sp. strain CC9605, have a highly conserved amino acid segment at the C-terminus, which is characterized by a very high content of charged amino acids (Figure 5). The corresponding region of the NitM proteins of β-cyanobacteria, including the one from *Synechococcus* sp. strain PCC 7002, showed no sequence conservation. These findings suggested that the presence or absence of the conserved C-terminal segment might have determined the functionally of the NitM proteins in the NA4 cells.

To examine the roles of the conserved C-terminal segment of the NitM protein of α-cyanobacteria, truncated *nitM* genes of *Synechococcus* sp. strain CC9311 and *Prochlorococcus marinus* strain MIT9313, lacking the 3’-terminal 63 nucleotides of the coding sequences, were fused to the *trc* promoter and introduced into the NA4 mutant to construct the NA412 and NA414 strains, respectively. Both NA412 and NA414 grew well on the medium containing 0.5 mM nitrite in the presence of 1 mM IPTG (Figure 2f,g) and took up low concentrations of nitrite from the liquid medium of pH 9.6 (Figure 4B). Nitrite uptake rates of the NA412 and NA414 mutants were calculated to be 21 and 38 µmol (mg Chl)^−1^·h^−1^, respectively. These results clearly indicated that the truncated NitM proteins were active in nitrite transport. The conserved C-terminal segment of the NitM proteins from α-cyanobacteria was thus deduced to inhibit the activity of the transporter.

## 3. Discussion

Nitrite transporters of the FNT family were previously characterized in the green alga *Chlamydomonas reinhardtii* (NAR1), the fungus *Aspergillus nidulans* (NitA) and the bacterium *Escherichia coli* (NirC). In the present study, the homolog of these proteins in the marine cyanobacterium *Synechococcus* sp. strain PCC 7002 was shown to be a nitrite-specific transporter having high affinity for the substrate (*K*_m_ = 8 µM) and named NitM. Data from transcriptome analysis in *Synechococcus* sp. strain PCC 7002 [25] show that expression of *nitM* is activated under the conditions of nitrogen deprivation and repressed by ammonium, suggesting that the gene is a functional gene in the cyanobacterium.

It should be noted that the cyanobacterial species isolated from marine and saline environments, including *Synechococcus* sp. strain PCC 7002, generally have the MFS-type nitrate/nitrite transporter NrtP as the sole transporter responsible for nitrate uptake [19]. While the ABC-type nitrate/nitrite transporter found in freshwater strains of cyanobacteria has high affinity for both substrates, allowing for simultaneous uptake of nitrate and nitrite, NrtP has much higher affinity for nitrate than for nitrite and hence preferentially takes up nitrate from the media containing the two substrates [26]. Thus, NitM would help the cyanobacterial strains carrying NrtP as the sole NRT species to take up nitrite simultaneously with nitrate. The presumed physiological role of NitM is supported by the fact that all but one of the 13 *nitM*-bearing cyanobacterial species with completely sequenced genomes carry NrtP as the sole NRT species.

When expression of *nitM* of *Synechococcus* sp. strain PCC 7002 was induced by IPTG, the NA401 mutant died on the nitrite-containing medium but not on the ammonium-containing medium (Figure 2, Row b). This indicates that overexpression of NitM was not harmful to the cells but excessive uptake of nitrite by the overexpressed NitM protein damaged the cells. Although the activity of the NrtP transporter is not regulated posttranslationally [26], the other cyanobacterial transporters known to have high-affinity for nitrite, *i.e.*, the ABC-type nitrate/nitrite transporter and the ABC-type cyanate/nitrite transporter, are tightly regulated at the post-translational level; Addition of ammonium to the medium completely inhibits the activity of the transporters [24,27,28,29,30,31]. The activity of nitrate reductase is also inhibited by ammonium in some species of cyanobacteria [26,28]. The inhibition of ABC-type nitrate/nitrite transporter, nitrate reductase, and ABC-type cyanate/nitrite transporter requires the P_II_ protein [29,31,32,33], the sensor of cellular nitrogen status, and is thought to allow for preferential utilization of ammonium over the other nitrogen sources, helping the cells to save energy under light-limited conditions, but it would also help the cyanobacterial cells to avoid excessive accumulation of nitrite.

Given the toxicity of excessive nitrite, the role of the C-terminal sequences conserved in most of the NitM proteins from α-cyanobacteria should also be discussed in terms of regulation of the transport activity. Although the “full-length” NitM proteins from *Synechococcus* sp. strain CC9311 and *Prochlorococcus* *marinus* strain CC9313 are inactive in the cells of *S. elongatus* strain PCC 7942 (Figure 4), they are unlikely to be permanently inactive in the cells of the α-cyanobacterial species; The gene is strongly conserved in the α-cyanobacterial species that have the capacity of nitrite assimilation and is tightly linked with the *nirA* gene for nitrite reductase, presumably forming an operon. Also nitrite concentration in the ocean is at most 0.6 µM and hence active uptake of nitrite would be essential for the cells to assimilate nitrite. These considerations suggest the presence of a mechanism to activate the α-cyanobacterial NitM protein by interacting with its C-terminal regulatory domain. The regulatory mechanism should be reversible, because photosynthesis in the ocean is not necessarily limited by nitrogen and in such cases, unregulated uptake of nitrite should be avoided. Further studies are needed to elucidate the molecular mechanism of regulation of NitM *in vivo*.

The absence of the C-terminal regulatory domain in the NitM proteins from *Synechococcus* sp. strain PCC 7002 and *Synechococcus* sp. strain CC9605, on the other hand, raises another question as to the regulation of nitrite uptake in these strains. In *Synechococcus* sp. strain PCC 7002 and the other β-cyanobacteria having the *nitM* gene, the gene is not linked with other genes involved in nitrite or nitrite assimilation and hence, expression of *nitM* may be tightly regulated at the step of transcription. By contrast, differential regulation of *nitM* and *nirA* is unlikely in *Synechococcus* sp. strain CC9605 because *nitM* is linked with *nirA* as in the other α-cyanobacterial strains. Thus, *Synechococcus* sp. strain CC9605 may have no opportunity to encounter excessive nitrite under natural conditions. It may be interesting to note that the cells of *Synechococcus* sp. strain CC9605 we obtained from Provasoli-Guilland National Center carried a mutation in *nitM* causing replacement of a strongly conserved Phe at position 65 with Leu, which needed to be fixed before the characterization of the gene in *S. elongatus* strain PCC 7942 (see Materials and Methods). The mutated *nitM* gene did not confer nitrite transport activity on the NA4 mutant of *S. elongatus*, indicating that the conserved Phe is important for the function of the protein. These results suggest that *Synechococcus* sp. strain CC9605 cells suffered from excessive nitrite during cultivation under the laboratory conditions, allowing for accumulation of the cells carrying the mutation in the transporter gene.

## 4. Materials and Methods

### 4.1. Strains and Growth Conditions

The nitrite-transport-less cyanobacterial mutant (NA4), which was constructed from the small-plasmid-cured (SPc) derivative of the β-cyanobacterium *S. elongatus* strain PCC 7942 [34] by deleting the *nrtABCD* genes encoding the ABC-type nitrate/nitrite transporter and the *cynABD* genes encoding the ABC-type cyanate/nitrite transporter (NA4) [24] was used as the host for heterologous expression of the FNT family protein of marine species of cyanobacteria (Table 1). Cells were grown photoautotrophically as described previously [35]. Ammonium-containing medium, nitrite-containing medium, and nitrate-containing medium were prepared by the addition of 3.75 mM (NH_4_)_2_SO_4_, 0.5 mM NaNO_2_, and 60 mM KNO_3_, respectively, to a nitrogen-free medium obtained by the modification of BG11 medium [36] as described previously [35]. Solid media were prepared by adding 1.5% Bacto agar (Difco) to the liquid media. Media were buffered with 20 mM HEPES-KOH (pH 8.2) or 10 mM 2-(*N*-cyclohexylamino)ethanesulfonic acid (CHES)-KOH (pH 9.6). When appropriate, kanamycin and chloramphenicol were added to the media at 25 and 6 μg·mL^−1^, respectively.

**Table 1:** Cyanobacterial strains and plasmids used.

Strain or plasmid	Relevant characteristics	Reference
Strains		
SPc	*Synechococcus elongatus* strain PCC 7942 cured of the pUH24 plasmid, wild type	[34]
NA4	SPc ∆*nrtABCD* ∆*cynABD*::Cm^R^, lacking the genes encoding an ABC-type nitrate/nitrite transporter and an ABC-type cyanate/nitrite transporter	[24]
NA41	NA4 harboring pSE1	[24]
NA401	NA4 harboring pS7002	This study
NA402	NA4 harboring pS9311	This study
NA403	NA4 harboring pS9605	This study
NA404	NA4 harboring pP9313	This study
NA412	NA4 harboring pS9311∆	This study
NA414	NA4 harboring pP9313∆	This study
Plasmids		
pSE1	Km^R^, *Synechococcus* shuttle expression vector	[23]
pS7002	pSE1 derivative encoding *nitM* of *Synechococcus* sp. strain PCC 7002	This study
pS9311	pSE1 derivative encoding *nitM* of *Synechococcus* sp. strain CC9311	This study
pS9605	pSE1 derivative encoding *nitM* of *Synechococcus* sp. strain CC9605	This study
pP9313	pSE1 derivative encoding *nitM* of *Prochlorococcus marinus* strain MIT 9313	This study
pS9311∆	pSE1 derivative encoding *nitM* of *Synechococcus* sp. strain CC9311 lacking the C-terminal region (21 a.a.)	This study
pP9313∆	pSE1 derivative encoding *nitM* of *Prochlorococcus marinus* strain MIT 9313 lacking the C-terminal region (21 a.a.)	This study

### 4.2. Expression of Plasmid-Encoded Proteins in S. elongatus Strain PCC 7942

A shuttle expression vector (pSE1) [23] was used for the expression of cloned genes in the NA4 mutant of *S. elongatus* strain PCC 7942. 0.8-kbp DNA fragments carrying the coding regions of the genes encoding the FNT family protein were amplified by PCR from the cells of *Synechococcus* sp. strain PCC 7002, *Synechococcus* sp. strain CC9605 (CCMP3075), and *Prochlorococcus* *marinus* strain MIT9313 (CCMP2773) and the chromosomal DNA of *Synechococcus* sp. strain CC9311. Cells of *Synechococcus* sp. strain CC9605 were obtained from Provasoli-Guilland National Center for Culture of Marine Phytoplankton. The forward primers used for the amplification of the genes of *Synechococcus* sp. strain PCC 7002, *Synechococcus* sp. strain CC9311, and *Synechococcus* sp. strain CC9605 carried mismatches with the respective genomic sequences, which were introduced to create an NcoI recognition site at the translation start site without changing the encoded amino acid sequences. The reverse primers used for the amplification of these genes carried an XbaI recognition site immediately downstream of the termination codon. The forward primer used for the amplification of the gene of *Prochlorococcus marinus* strain MIT9313 carried an MfeI recognition site, which was created at the translation start site without changing the encoded amino acid sequence. The reverse primer for the amplification of the *Prochlorococcus* gene carried a BamHI recognition site immediately downstream of the termination codon. The internal NcoI recognition sequence CCATGG of the gene of *Synechococcus* sp. strain CC9605 was changed to CGATGG without changing the encoded amino acid sequence by overlapping PCR [37]. Since the gene sequence of the *Synechococcus* sp. strain CC9605 cells obtained from Provasoli-Guilland National Center had a C-to-A mutation at the 195th position of the ORF, changing the Phe residue at position 65 to a Leu residue (F65L), overlapping PCR [37] was employed to restore the published sequence of the gene. For expression of C-terminally truncated forms of the FNT family proteins from *Synechococcus* sp. strain CC9311 and *Prochlorococcus marinus* strain MIT9313, the reverse primers carrying a SpeI recognition site (ACTAGT) were used for PCR amplification of the genes to introduce a termination codon at nucleotide positions 823 and 826 for the genes of *Synechococcus* sp. strain CC9311 and *Prochlorococcus marinus* strain MIT9313, respectively. The PCR-amplified genes and their derivatives were digested with a combination of NcoI/XbaI, MfeI/BamHI or NcoI/SpeI and cloned between the NcoI and XbaI sites or the NcoI and BamHI sites of pSE1. The resulting plasmids were introduced into cells of the *S. elongatus* NA4 mutant after verification of the nucleotide sequences.

### 4.3. Measurements of Nitrite Uptake

Cells grown in nitrate (60 mM)-containing medium (pH 8.2) were washed with the basal medium supplemented with 10 mM KHCO_3_, 5 mM K_2_CO_3_, and 10 mM CHES-KOH (pH 9.6) and suspended in the same medium. The reaction was started by addition of NaNO_2_ to the cell suspensions, which were kept at 30 °C in the light (100 μE m^−2^·s^−1^). Aliquots were withdrawn from the cell suspensions at 5-, 10-, or 15-min intervals, and after immediate centrifugation for 60 s at 15,000× *g* to sediment the cells, the nitrite concentration in the supernatant was determined.

### 4.4. Other Methods

Nitrite was determined as described by Snell and Snell [38]. Chl was determined according to Mackinney [39]. Manipulations and analyses of DNA were performed according to standard protocols.

## Supplementary Materials

Supplementary materials can be accessed at: http://www.mdpi.com/2075-1729/5/1/432/s1.
