# Supplementary material for: Functional Characterization of the FNT Family Nitrite Transporter of Marine Picocyanobacteria
Source: Life (Basel). 2015 Feb 9;5(1):432–46. doi: 10.3390/life5010432 (PMC4390861; doi:10.3390/life5010432)
Supplement: Supplementary file 1 [file life-05-00432-s001.pdf]

Supplementary Materials

Table S1. Distribution of the *narB*, *nirA*, and *nitM* genes in cyanobacteria.

Strain	*narB*	*nirA*	*nitM*
**α-cyanobacteira**			
Cyanobium gracile PCC6307	+	+	-
Synechoccocus sp. WH8102	+	+	-
Synechococcus sp. CC9605	+	+	+
Synechococcus sp. CC9902	+	+	+
Synechococcus sp. CC9311	+	+	+
Synechococcus sp. WH7803	+	+	+
Synechococcus sp. RCC307	+	+	+
Prochlorococcus marinus NATL1A	-	+	+
Prochlorococcus marinus NATL2A	-	+	+
Prochlorococcus marinus MIT9303	-	+	+
Prochlorococcus marinus MIT9313	-	+	+
Prochlorococcus sp. MIT 0801	-	+	+
Prochlorococcus marinus MIT9211	-	-	-
Prochlorococcus marinus SS120	-	-	-
Prochlorococcus marinus MED4	-	-	-
Prochlorococcus marinus MIT9515	-	-	-
Prochlorococcus marinus MIT9215	-	-	-
Prochlorococcus marinus MIT9301	-	-	-
Prochlorococcus marinus MIT9312	-	-	-
Prochlorococcus marinus AS9601	-	-	-
Prochlorococcus sp. MIT 0604	-	-	-
**β-cyanobacteira**			
Gloeobactor violaceus PCC742	+	+	-
Gloeobacter sp. JS	+	+	-
Synechococcus sp. (Yellowstone a')	+	+	-
Synechococcus sp. (Yellowstone b')	+	+	-
Thermosynechococcus elongates BP1	+	+	-
Thermosynechococcus sp. NK55	+	+	-
Pleurocapsa sp. PCC7327	+	+	-
Microcystis aeruginosa NIES-843	+	+	-
Cyanothece sp. PCC7425	+	+	-
Synechocystis sp. PCC6803	+	+	-
Synechococcus sp. PCC 7502	+	+	-
Synechococcus elongatus PCC6301	+	+	-
Synechococcus elongatus PCC7942	+	+	-
Anabaena sp. PCC7120	+	+	-
**β-cyanobacteira continued**			
Anabaena variabilis ATCC29413	+	+	-
Pseudoanabaena sp. PCC7367	+	+	-
Gloeocapsa sp. PCC7428	+	+	-
Camaesiphon minitus PCC6605	+	+	-
Cyanobacterium stanieri PCC 7202	+	+	+
Cyanobacterium aponinum PCC10605	+	+	-
Calothrix sp. PCC 6303	+	+	-
Nostoc sp. PCC7524	+	+	-
Cyanothece sp. PCC7424	+	+	-
Cyanothece sp. PCC8801	+	+	-
Cyanothece sp. PCC8802	+	+	-
Arthrospira platensis NIES-39	+	+	-
Cyanothece sp. PCC 7822	+	+	-
Stanieria cyanosphaera PCC 7437	+	+	-
Chroococcidiopsis thermalis PCC 7203	+	+	-
Geitlerinema sp. PCC 7407	+	+	-
Microcoleus sp. PCC 7113	+	+	-
Crinalium epipsammum PCC 9333	+	+	-
Oscillatoria nigro-viridis PCC 7112	+	+	-
Nostoc sp. PCC 7107	+	+	-
Nostoc punctiforme ATCC29133	+	+	-
Synechococcus sp. PCC 6312	+	+	-
Oscillatoria acuminata PCC6304	+	+	-
Anabaena sp. 90	+	+	-
Anabaena cylindrica PCC7122	+	+	-
Cylindrospermum stagnale PCC7417	+	+	-
Calothrix sp. PCC 7507	+	+	-
Rivularia sp. PCC 7116	+	+	-
Halothece sp. PCC 7418	+	+	+
Dactylococcopsis salina PCC 8305	+	+	+
Cyanothece sp. ATCC51142	+	+	-
Trichodesmium erythraeum IMS101	+	+	-
Leptolyngbya sp. PCC7376	+	+	-
Acaryochloris marina MBIC11017	+	+	-
Synechococcus sp. PCC7002	+	+	+
Anabaena azollae 0708	-	-	-
Cyanobacterium_UCYN-A, 713887	-	-	-
